# Neurocognitive Outcomes and School Performance in Solid Tumor Cancer Survivors Lacking Therapy to the Central Nervous System

**DOI:** 10.3390/jpm5020083

**Published:** 2015-04-10

**Authors:** Caroline Mohrmann, Jennifer Henry, Marnie Hauff, Robert J. Hayashi

**Affiliations:** Washington University School of Medicine, 1 Children’s Place, Campus Box 8116 Saint Louis, MO 63110, USA; E-Mails: Henry_Je@kids.wustl.edu (J.H.); Hauff_M@kids.wustl.edu (M.H.); Hayashi_R@kids.wustl.edu (R.J.H.)

**Keywords:** young children, solid tumors, neurocognitive, school performance, neuropsychological testing

## Abstract

School performance in patients who have received therapy for childhood cancers has been studied in depth. Risk factors have historically included cranial radiation, intrathecal chemotherapy, and high doses of chemotherapy, including methotrexate and cytarabine. Leukemia and brain tumor survivors who receive such therapy have been the primary focus of this area of investigation. Extracranial solid tumor cancer patients lacking such risk factors have historically been expected to have normal school performance. We examined the medical records of 58 young pediatric extracranial solid tumor patients who lacked CNS-directed therapy or other known risk factors for cognitive impairment to evaluate the incidence of reported difficulties or abnormalities in neuropsychological testing. Thirty-one percent of patients were found to have at least one reported difficulty or abnormality. Of note, 34% of patients with Wilms tumor possessed difficulties compared to 23% of patients with other extracranial solid tumors. Extracranial solid tumor cancer survivors without known risk factors for school performance difficulties appear to have a higher incidence of problems than expected.

## 1. Introduction

Neurocognitive changes after the completion of chemotherapy for childhood cancers can have a significant effect on the quality of life of survivors [1,2,3]. Childhood cancer survivors as a whole are more likely to utilize special education services than their siblings, especially those diagnosed at a young age, but certain survivors have been targeted for their especially high rate of neurocognitive dysfunction and education needs [4]. Survivors of brain tumors are known to have neurocognitive changes after therapy due to neurosurgical interventions, cranial radiation, and chemotherapy directed to the central nervous system (CNS). This functional impairment can be an imposing burden for these patients, as they are unable to complete levels of higher education, obtain employment, and live independently [5]. Survivors of childhood acute lymphoblastic leukemia (ALL), especially those with a history of cranial radiation, also experience worse school performance than their peers [6]. A study from the Netherlands revealed that, when compared to their siblings, survivors of ALL were more often in a special education program. Survivors also achieved a lower level of secondary education [7]. Young age at diagnosis of ALL has often been found to increase the risk for neurocognitive deficits [7,8,9]. Some studies evaluate neurocognitive effects in patients with a history of extracranial solid tumors, but do not limit their sample to patients treated with non-CNS directed therapies. Extracranial solid tumor and leukemia survivors as a whole perform below the level of their peers in the areas of task efficiency, memory, and emotional regulation [8]. The groups most greatly affected are females, patients treated at a very young age, and those who were treated with cranial radiation [8].

Neurodevelopmental functioning in very young children with extracranial solid tumors and leukemia treated with chemotherapy alone were found to perform worse on motor and mental assessments, as well as a general developmental assessment [10]. The aforementioned study did not separate those who had received CNS-direct therapy from those who did not. The study could not include reports of school performance due to the age at evaluation being prior to the age of school entry. Overall, there is a paucity of literature addressing the neurocognitive status of children treated at a young age, despite concerns that their treatment occurred at a crucial time in their social and cognitive development. These young children can become quite ill during therapy, which prohibits important developmental play or early education. Tumors with a high-incidence at a young age, such as Wilms Tumor, Neuroblastoma, and Germ Cell Tumors, often do not require treatment to the CNS, yet the impact of systemic chemotherapy alone (plus or minus local radiation) has not been studied in depth. Existing literature on neurocognitive functioning after therapy for childhood cancer often includes those with brain tumors or CNS-direct therapies, making it difficult to appreciate the neurocognitive status of children treated without CNS-direct therapies. The impact of treatment for these children would be important to understand in order to provide support, as well as education, to their families in terms of their specific education needs. We therefore sought to determine whether these children were negatively impacted by their treatment.

## 2. Subjects and Methods

### 2.1. Subjects

Children treated for malignant extracranial solid tumors at St. Louis Children’s Hospital and the Washington University School of Medicine, excluding those who received any CNS-directed therapies, were considered for the study. A total of 290 patients were identified from the population followed in our Late Effects Clinic, which cares for childhood cancer survivors two years or more after the completion of therapy. We were particularly interested in patients who received therapy prior to starting school to assess the impact of therapy on their subsequent performance. Patients undergoing full chart review met the following inclusion criteria: successful completion of therapy for Wilms Tumor, Rhabdomyosarcoma, Neuroblastoma, Hepatoblastoma, or other malignant extracranial solid tumor, and diagnosed at age 6 or earlier. Patients were excluded if there were risks known to be associated with school performance difficulties including: previous exposure to intrathecal chemotherapy, received radiation to any site on the head, diagnosis of a brain tumor, experienced vision loss or hearing loss, had a genetic or preexisting condition that predisposes to neurocognitive deficiencies, or were known to have such difficulties prior to their cancer diagnosis. To maximize the size of the population, we included patients with whom we no longer have an existing treating relationship.

### 2.2. Methods

Eligible patients’ charts were screened for reported incidence of academic difficulties or neurocognitive deficits. As a retrospective study, all data were extracted from preexisting chart records. All incidences of subjective school difficulty as reported by patient, parent or guardians were recorded. Subjective school difficulty was excluded if it was deemed to be emotional or psychosocial in nature, rather than neurocognitive in nature. Examples of subjective school difficulties reported include “he has developed some issues with decreased school performance”, “he is having some school problems, especially regarding math and science,” “he was in a special program for reading assistance”, and “she is in a class where students receive extra help”. Objective data were also recorded, including: Failed grade, use of a 504 Plan, use of an Individualized Education Program (IEP), or deficiencies on neuropsychological testing (NPT) as determined by a neuropsychologist.

Demographic data collected included chemotherapy administered, sites of radiation, years from treatment termination, current age, gender, race, maximum educational grade level, presence of a genetic condition, and other medical conditions. Consent was not obtained as information was preexisting and available from the medical records; no further participation from the subjects was required. Subjects’ identity was coded to optimize confidentiality. The medical center’s institutional review board approved all procedures.

### 2.3. Data Analysis

This descriptive analysis consisted of means and standard deviations for continuous variables and frequencies for categorical variables cited above. There was no comparable control population that has been followed serially with school performance monitored available to these investigators. Chi-square test and Fisher’s exact test were used to determine the association of risk factors and school difficulties. All results were considered statistically significant at the *p* < 0.05 level.

## 3. Results

Patient characteristics are shown in Table 1. There were a total of 58 eligible patients who underwent chart review. The majority of the patients were less than or equal to two years of age at diagnosis (60.3%), with a mean age of 2.34 years. Age at time of study ranged from 1 to 30.4 years, with average age of 12.5 years. The majority of patients were survivors of Wilms Tumor (*n* = 32), followed by Neuroblastoma (*n* = 16), Rhabdomyosarcoma (*n* = 6), Ewing’s Sarcoma (*n* = 2), Germ Cell Tumor (*n* = 1), and Triton Tumor (*n* = 1).

**Table 1:** Patients’ clinical and demographic characteristics.

Patient Characteristics	Total Number (58)	Percentage (%)
**Age at Diagnosis (Years)**		
0–2.99	35	60.3%
3–4.99	20	34.4%
5–6	3	5.2%
**Current Age (Years)**		
0–5.99	3	5.2%
6–10.99	25	43.1%
11–15.99	16	27.6%
16–20.99	11	18.9%
>21	3	5.2%
**Gender**		
Male	27	46.6%
Female	31	53.4%
**Race**		
African American	7	12.1%
Caucasian	50	86.2%
Hispanic/Latino	1	1.7%
**Diagnosis**		
Wilms Tumor	32	55.2%
Rhabdomyosarcoma	6	10.3%
Neuroblastoma	16	27.6%
Ewing’s	2	3.4%
Germ Cell Tumor	1	1.7%
Triton Tumor	1	1.7%

Overall, 31% of patients were found to have at least one self-reported subjective difficulty in school, objective difficulty such as failed grade, or abnormal finding(s) on NPT in their medical record, indicating some level of academic difficulty. The incidences of subjective and objective difficulties occurring by diagnosis are displayed in Table 2. The most common finding was subjective school difficulties as reported by the parent or student, with a total of 17 patients (29.3%). Our largest population, survivors of Wilms Tumor, experienced a high incidence of academic difficulties with 11 of the 31 patients having at least one reported difficulty (34.3%). The highest incidence was reported amongst rhabdomyosarcoma survivors, with three of the six patients having least one difficulty (50%). Six patients (10.3%) were noted to have a formal plan for academic assistance in place at school, such as a 504 Plan or IEP. When reviewing demographic data and treatment information, there were no statistically significant correlations between reported difficulties and therapy history. We also failed to find correlation between academic difficulties and age, race, or gender.

**Table 2:** Academic difficulties experienced by young extracranial solid tumor patients

Type of Academic Difficulty	Total (*n* = 58)	Wilms Tumor (*n* = 32)	Rhabdo-myosarcoma (*n* = 6)	Neuroblastoma (*n* = 16)	Ewing’s Sarcoma (*n* = 2)	Germ Cell Tumor (*n* = 1)	Triton Tumor (*n* = 1)
Subjective School Difficulties	17 (29.3%)	11 (34.3%)	3 (50%)	2 (12.5%)	0 (0%)	0 (0%)	1 (100%)
Failed Grade	3 (5.1%)	0 (0%)	1 (16.6%)	2 (12.5%)	0 (0%)	0 (0%)	0 (0%)
504 Plan	2 (3.4%)	2 (6.3%)	0 (0%)	0 (0%)	0 (0%)	0 (0%)	0 (0%)
IEP	4 (6.9%)	2 (6.3%)	1 (16.6%)	0 (0%)	0 (0%)	0 (0%)	1 (100%)
Deficiencies on NPT	8 (16.6%)	5 (15.6%)	0 (0%)	2 (12.5%)	0 (0%)	0 (0%)	1 (100%)
Total # with Academic Difficulties	18 (31%)	11 (34.4%)	3 (50%)	3 (18.8)	0 (0%)	0 (0%)	1 (100%)

Interestingly, with 29.3% of patients reporting school difficulties, only nine patients (15.5%) were referred for and completed neuropsychological testing. Of the nine patients tested, eight had deficiencies noted during their exam (89%). Further analysis of this sub-group reveals the majority of the patients had a primary diagnosis of Wilms Tumor (66%). All patients in this group had received chemotherapy, as well as surgical resection. This group was also more likely to have support in place at school (55%), compared to those without neuropsychological testing (2%).

## 4. Discussion

### 4.1. Implications for Practice

Survivors of extracranial solid tumors in early childhood appear to have a higher than expected incidence of reported academic difficulties. In this report the incidence of subjective school difficulties was higher than would be expected for children without CNS-directed therapies, with 31% of patients reporting at least one school difficulty. Documentation of the extent of the difficulty was limited, as only 15.5% of the patients had formal NPT, although 89% of those tested demonstrated significant deficiencies. According to the U.S. Department of Education for the academic school year 2008–2009, 8% of students received services for specific learning disabilities, intellectual disabilities, other health impairments, or developmental delays. Although our population has a similar incidence of special educational services, with 10.3% of our population having an IEP or 504 Plan, it is likely that more children would benefit from extra academic assistance. A contributing factor is likely our lack of uniform referral for neuropsychological evaluations of this population. Of the 31% of patients in our sample that reported difficulties, over half were not referred for neuropsychological testing. We subsequently found patients who had NPT were much more likely to have academic support initiated, with only one patient having a formal plan in place without NPT records at this institution.

Approximately half of our patients with reported difficulties were not referred for NPT. This raises the question as to what prompts a provider to initiate such a referral. Our review shows that reports of subjective difficulties do not prompt enough concern in all providers to make a referral for NPT. There are several reasons that could potentially account for this discrepancy. Providers may not appreciate the importance of formal NPT in this population, or not believe that this population lacking known risk factors would be at risk for school performance difficulties. Parents may resist formal testing due to the expense or the stigma of the identification of a potential deficit. Testing may not be feasible due to lack of access to appropriate resources. Further investigation is needed to identify the obstacles for testing to identify patients who would benefit from additional services.

Our results are interesting in light of previous work by Buizer, who evaluated survivors of leukemia and Wilms Tumor in the Netherlands. The total score for school performance according to the School Performance Index for children with Wilms Tumor was not statistically different than controls, although 31% of patients with Wilms Tumor scored outside the normal range and only 17% of controls scored outside the normal range [11]. The tests used to measure neurocognitive difficulties also contrast our standard NPT that includes measures of executive functioning, as well as standard academic assessments. This skill is critical for childhood cancer survivors, since lower levels of executive functioning are related to worse educational and psychosocial outcomes [12,13].

It would be reasonable to have a low threshold for referral in order to characterize the difficulties the child is experiencing. Healthcare providers working in long-term follow-up clinics should be sensitive to the needs of this population in order to identify areas in which interventions will improve their academic outcomes and may also positively impact their psychosocial outcomes as adults.

### 4.2. Study Limitations

Our study has many limitations, most notably a small sample size from a single institution. With our limited size, it was not possible to determine if any treatment characteristics or demographic data were significant in relation to school performance. Since we excluded patients with vision or hearing loss, we had to exclude a great number of patients treated for Neuroblastoma, Retinoblastoma, and Hepatoblastoma. Patients with these conditions possess a high incidence of hearing loss due to treatments, including ototoxic agents such as cisplatin, and were excluded to avoid introducing another confounding variable into the analysis [14]. These children represent a population in which academic performance requires close monitoring.

We also did not include household income or parental educational level, variables which are known to impact a child’s academic performance [15]. We also lacked a control group that would have been helpful to establish if reported incidence varies among children without a cancer history, or possibly children with a cancer history who were treated at an older age. Our academic difficulties were all subjective; we lacked official academic records to validate if these subjective difficulties transcended the student’s school performance. Capture of all patients with academic difficulties was limited, as we cannot ensure that all subjective difficulties reported to the provider were documented in the medical record. Many would criticize using subjective data, and we recognize the limitations of such, but argue that patient, and family, reported outcomes documented in the medical record are important to clinical care. Despite these limitations, the high frequency of reported school difficulties warrants that extracranial solid tumor cancer patients treated at an early age may be at substantial risk for school difficulties; this includes even those who lack an obvious risk factor for neurocognitive toxicity from their therapy. Close monitoring and formal neurocognitive evaluations are needed to identify significant problems so that appropriate services are mobilized to optimize school performance.

## 5. Conclusions

Although young children treated for extracranial solid tumors without CNS-directed therapies are not usually targeted for neurocognitive assessment or intervention, it is possible that they are indeed at risk for experiencing cognitive challenges after the completion of therapy. Clinicians should heighten their awareness of this potential problem and obtain formal neurocognitive testing once difficulties are identified. Further research would be necessary to confirm our findings, especially in young cancer survivors also at risk for vision and/or hearing loss, and to evaluate effective interventions for young children completing chemotherapy in order to optimize their school performance.
